# Effect of Collagen Cross-Link Deficiency on Incorporation of Grafted Bone

**DOI:** 10.3390/dj7020045

**Published:** 2019-05-01

**Authors:** Suliman Mubarak, Nagasawa Masako, Farah A. Al-Omari, Hamaya Keisuke, Uoshima Katsumi

**Affiliations:** Division of Bio-Prosthodontics, Graduate School of Medical and Dental Sciences, Faculty of Dentistry, Niigata University, 2-5274 Gakkocho-dori, Chuo-ku, Niigata 951-8514, Japan; mubarakoo86@hotmail.com (S.M.); omari@dent.niigata-u.ac.jp (F.A.A.-O.); hamaya@dent.niigata-u.ac.jp (H.K.); fish@dent.niigata-u.ac.jp (U.K.)

**Keywords:** collagen cross-links, bone quality, host bone, graft, Healing

## Abstract

Bone matrix collagen, is one of the major contributors to bone quality. No studies have examined how bone quality affects the results of bone transplantation. Collagen cross-links (CCL) are the key factor in collagen properties. The purpose was to investigate the influences of CCL for both grafted bone and recipient site bone on the success of bone augmentation. Four-week-old male Wister rats (*n* = 54) were divided into control and test groups. Control and test groups equally sub-divided into donors and recipients. An additional six rats were used to characterize bone at day zero. Test groups received 0.2% beta-aminoproperionitrile (BAPN) for 4 weeks as CCL inhibitor. Animals were further divided into donor and recipient groups. The transplanted bone chips integrated with host bone by 25% more in CCL-deficient animals compared to control. However, no difference in cortical thickness among all conditions. CCL-deficient transplanted bone did not show any extra signs of osteocyte apoptosis, while sclerostin expression was comparable to that in control. The host periosteum of CCL-deficient animals showed higher cellular activity, as well as higher bone quantity and osteoclast activity. Collagen cross-links deficiency in host bone might accelerate the incorporation of grafted bone. effect. Incorporation of the bone grafts appears to depend mainly on host condition rather than graft condition.

## 1. Introduction

The success of implant therapy is directly related to the surrounding bone bed. Sometimes, bone augmentation is required to increase the volume of bone at the proposed site of implant installation. Bone grafting became an essential part of dental practice such as pre-prosthetic bone augmentation, jaw reconstruction, and sinus lifting. The bone for transplantation could be harvested from either local or distant sites [1,2,3]. In any cases, to achieve successful bone augmentation, both grafted bone and host bone should meet certain criteria. The definition of success for bone transplantation is the union of transplanted bone with host bone after an orchestrated process of inflammation, osteogenesis, and remodeling [4], resulting in incorporation of the grafted bone. For this reason, the choice of donor site should be highly influenced by its biological quality [1]. However, while several studies have examined differences in prognosis according to the harvest site of grafted bone, no studies have examined how the quality of the grafted bone along with the host bone would affect the results of bone augmentation.

Collagen type 1 is known to be a major type of collagen in bone constituting around 90% of the organic component. Collagen cross-links are a key factor in determining collagen properties. Collagen cross-linking is directly related to bone mechanical properties [5,6,7,8,9] and is accordingly also thought to represent a major determinant of bone quality. The bone quality parameters examined in dentistry are shifting from the old concept of considering only bone mineral density to adding new concepts from microstructural evaluation [9]. This affirms the need to clarify the role of collagen cross-links in predicting the outcomes of bone surgery.

Beta-aminoproperionitrile (BAPN) is well documented as a potent inhibitor of lysyl oxidase (LOX) enzyme in vitro and in vivo [10,11,12,13]. The mechanism of BAPN action is through direct chemical binding and irreversible inhibition of LOX enzyme [14]. A previous report demonstrated that reduced collagen cross-links using BAPN in the matrix could directly affect the behavior of bone cells in vitro [15]. However, whether collagen cross-link deficiency in vivo would affect bone tissue-related cells has yet to be clarified.

The purpose of this study was to investigate the influences of collagen cross-link deficiency in both grafted bone and recipient-site bone on the success of bone augmentation. Based on previous findings of accelerated bone cells activity at the state of low collagen cross-links [16], our hypothesis was that collagen cross-link deficiency might accelerate bone formation, resulting in greater graft incorporation with host bone. The second objective was to clarify whether transplanted graft or host bone is more important for the incorporation of bone graft.

## 2. Materials and Methods

### 2.1. Collagen and Animal Models

**Figure 1:**
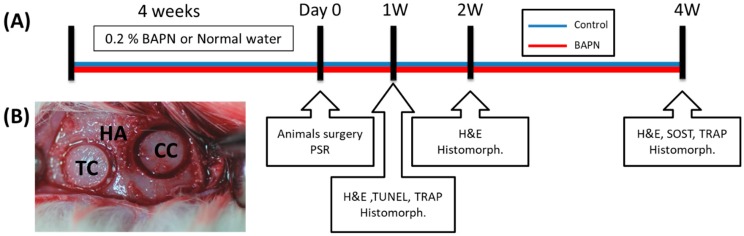
Schematic representation of the timeline of the experiment. (**A**) Control and beta-aminoproperionitrile (BAPN) (Test) groups and the accompanying investigation according to each time point; (**B**) Calvaria at the time of surgery. HA, host animal calvaria of either control or BAPN; CC, control chip; TC, BAPN chip.

To produce a rat model of deficient collagen cross-links, BAPN (Tokyo Chemical Industry, Tokyo, Japan) was used as a potent LOX enzyme inhibitor. Four-week-old male Wistar rats (*n* = 54) were recruited and randomly assigned to each experimental condition. The Test group (*n* = 24) received ad libitum access to drinking water containing 0.2% BAPN for a period of 4 weeks. Control rats (*n* = 24) received normal water. Each of the control and test groups was equally sub-divided into donor and recipient subgroups. The remaining six rats were used to characterize bone collagen at day zero. The recipient groups received control and test bone chips, as seen in Figure 1. All animal experiments and related procedures were approved by the Animal Care and Ethics Committee at Niigata University (approval #27-300-2.15/10/2015).

### 2.2. Animal Surgery

On the day of surgery, anesthesia was induced using sevoflurane volatile solution (Pfizer and Mylan, Canonsburg, PA, USA) inside a breathing chamber. Anesthesia was maintained by intraperitoneal injection of 8% trichloroacetaldehyde monohydrate (Wako Pure Chemical Industries, Osaka, Japan). Vertical incision through skin and muscles was performed over the calvaria. The periosteum was elevated, and two 5-mm-diameter bone chips were harvested by trephine bur under copious irrigation, then immediately transferred to recipient animals. Sites were stitched by interrupted sutures. Rats were sacrificed at three designated time points: One, two, and four weeks after operation (Figure 1) using a CO_2_ inhalation chamber. Samples were harvested and immersed in 10% formalin solution and changed every day for three days.

### 2.3. Histology

Ethylenediaminetetraacetic acid (EDTA) 10% was used to decalcify samples over the course of 4 weeks. Samples were then dehydrated in an alcohol series and embedded in a paraffin block. Coronal sections (5 µm) were made using a microtome (Yamato Koki, Asahi, Saitama, Japan). Samples were deparaffinized and stained using hematoxylin and eosin (HE) for baseline assessment. Picrosirius red stain (PRS) was applied according to the protocol defined by Junqueira et al. [17] for assessing collagen fibers. The orientation of collagen fibers was observed under a polarized lens for PRS-stained samples. Mature collagen fibers were seen as greenish-yellow, while immature collagen fibers were red in color. A tartrate-resistant acid phosphatase (TRAP) kit (Wako Pure Chemical Industries, Osaka, Japan) was used according to the instructions from the manufacturer to assess osteoclast staining. Apoptotic activity was detected using terminal deoxynucleotidyl transferase dUTP nick end labeling (TUNEL) staining kit (in situ Apoptosis Detection Kit—ab206386; Cambridge, MA, USA).

### 2.4. Histomorphometric Analysis

From the histological sections we measured the following parameters; Bone union (by new bone bridging between the transferred bone and host bone), and Cortical thickness (Ct.Th) was measured by selecting 20 arbitrary lines from the periosteal side to the meningeal side for each bone chip. Bone area (B.Ar), which includes newly formed bone and marrow spaces, and defect closure ratio (D.C) were measured. Measurements were made in accordance with Sohn et al. and Dempster and Compston et al. [18,19]. ImageJ software (NIH, Framingham, MA, USA) was used for histomorphometric analyses.

### 2.5. Immunohistochemical Analyses

Sclerostin expression by osteocytes was studied. The selected sections were incubated overnight with sclerostin primary antibody-polyclonal rabbit antibody to sclerostin (ab63097, Abcam, Cambridge, MA, USA) at 1/50 dilution. Subsequently, Goat anti-rabbit immunoglobulin G H&L horseradish peroxidase (ab205718, Abcam, Cambridge, MA, USA) was used as the secondary antibody and incubated for 1 h at 1/10,000 dilution. Antigens were retrieved by heat induction in a citric acid solution (pH 6). Nonspecific antigens were blocked using skim milk. Nonspecific reactions were blocked by hydrogen peroxide. Visualization was done by 3,3′-diaminobenzidine (DAB) and counterstained by methyl green. The ratio of sclerostin expressing osteocytes to all osteocytes in each section was calculated.

### 2.6. Statistical Analysis

Data were statistically analyzed using Microsoft Office (Excel 2010). Student’s *t*-test and Chi-square test were performed, and the *p* value was defined as (*p* < 0.05) to be statistically significant.

## 3. Results

### 3.1. Characterization of Collagen Structure

**Figure 2:**
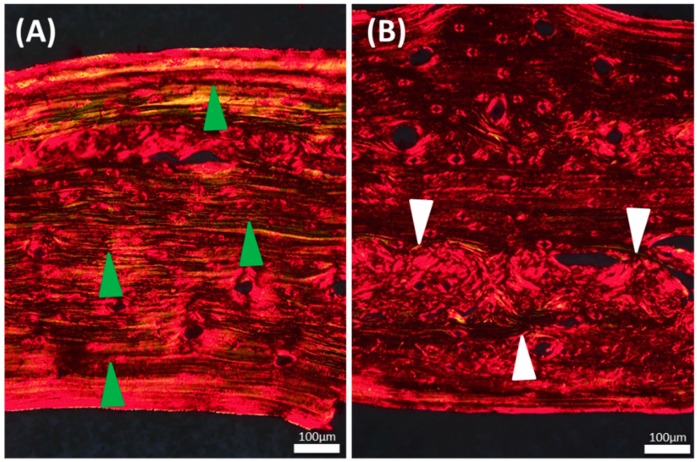
Characterization of collagen structure by the picrosirius red stain of calvaria in coronal cross-sections in which the bottom of the section is the meningeal side. Mature collagen fibers stain green/yellow, while immature collagen fibers stain red/orange. (**A**) Control group shows uniform collagen fibers running parallel to each other, as indicated by green arrowheads; (**B**) BAPN group shows fewer mature fibers and more immature collagen tissue as indicated by white arrowheads. Magnification, ×20.

We characterized collagen fibers by PRS at the beginning of the experiment to determine the effect of BAPN on collagen fiber maturation. The control group showed a greater frequency of uniform, parallel collagen fibers. In contrast, the BAPN group showed a greater frequency of immature collagen fibers in newly formed bone. Even in the BAPN group, uniform collagen fibers were observed in the bone area that seemed to have been formed prior to BAPN administration (Figure 2).

### 3.2. Histological and Immunohistochemical Analyses of Transferred Bone Chips

**Figure 3:**
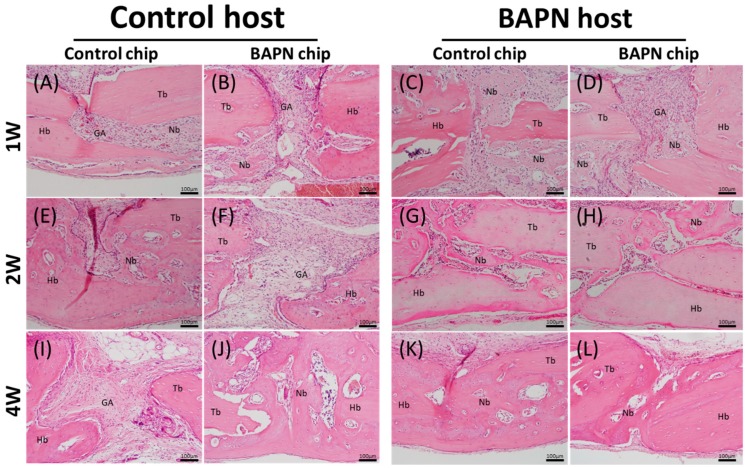
Hematoxylin- and eosin-stained microscopic images for incorporation in the gap area of bone bridging between host bone and transplanted bone chips. (**A**–**D**) At Week 1, in both groups, new bone was observed with the presence of blood vessels. BAPN hosts show more new bone formation inside the gaps compared to control hosts; (**E**–**H**) At Week 2, BAPN shows more bone bridging than control; (**I**–**L**) By Week 4, all BAPN hosts bridge the gaps with mineralized bone more often than controls. GA: Gap area; Hb: Host bone; Tb: Transplanted bone; Nb: New bone. Magnification, ×20.

HE staining of BAPN host bone showed a higher frequency of bone formation in gaps between host bone and transplanted bone. This applied to both control and BAPN chips (Figure 3), and indicated earlier bone formation in BAPN hosts (Figure 3C,D) compared to control hosts (Figure 3A,B) regardless of the type of transplanted chip. By Week 4, 75% of investigated sections showed gap closure in control host rats (Figure 3I,J) and 100% of investigated sections showed gap closure in BAPN host rats (Table 1). These percentages were equal for control and BAPN transplanted chips in both hosts (Table 1). The bone formation (i.e., bone union) between host and transferred bone for BAPN host was significantly higher by 25% higher than the control group (*p* = 0.012).

**Figure 4:**
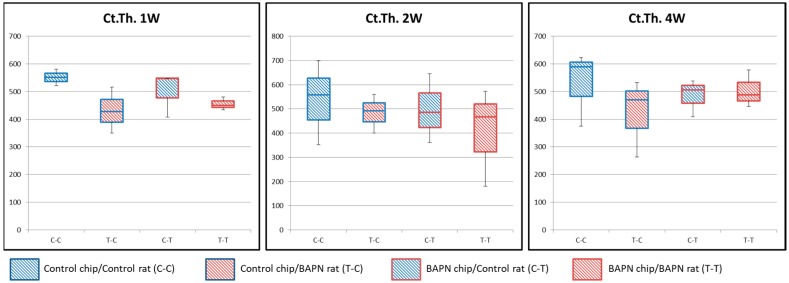
Cortical thickness of transplanted bone chips. No significant difference in cortical thickness is seen among all conditions.

Bone formation took place underneath transplanted bone chips on the meningeal side. Therefore, to investigate the effect of transplanted bone chip structure on new bone formation, cortical thickness of the transplanted chip was observed by measuring the distance from the periosteal side to the meningeal side under all conditions (Figure 4). No significant differences were seen among all groups.

**Figure 5:**
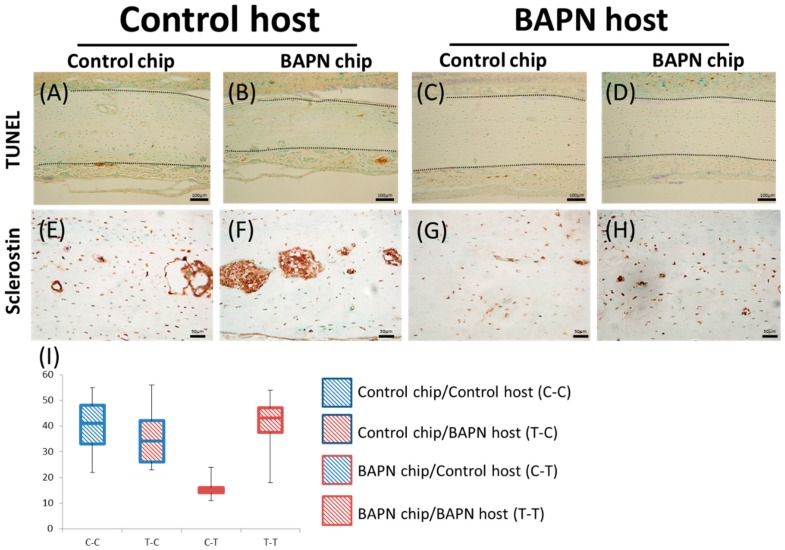
Histological findings of TUNEL and sclerostin staining of transplanted bone chips. (**A**–**D**) Histologically, no difference is seen in apoptosis as represented by TUNEL staining among all chips; (**E**–**H**) Histological expression of sclerostin does not show any difference; (**I**) Ratio of osteocytes expressing sclerostin to osteocytes in histological sections. No significant difference is seen among all transplanted bone chips. Dotted line defines the transferred bone. Magnification, ×40.

No difference in the number of TUNEL-positive cells was evident among all transplanted bone chips. Both BAPN and control groups exhibited the same degree of apoptotic activity. Some remaining apoptotic activity was seen in newly developed bone endosteally below the transplanted bone chip under all conditions (Figure 5A–D). By Week 4, all bone chips were expressing sclerostin with no differences between conditions (Figure 5E–H).

Table 1 Percentage of bone union formation by bone tissue bridges between transplanted bone and host bone. Each number represents the percentage of positive (i.e., union) sections out of all sections investigated. Regardless of transferred bone condition, control hosts versus BAPN host had 75 and 100% respectively of bone bridging (i.e., unions). The non-union of control and BAPN host was 25 to 0% respectively.

### 3.3. Periosteum Surrounding Transferred Bone

**Figure 6:**
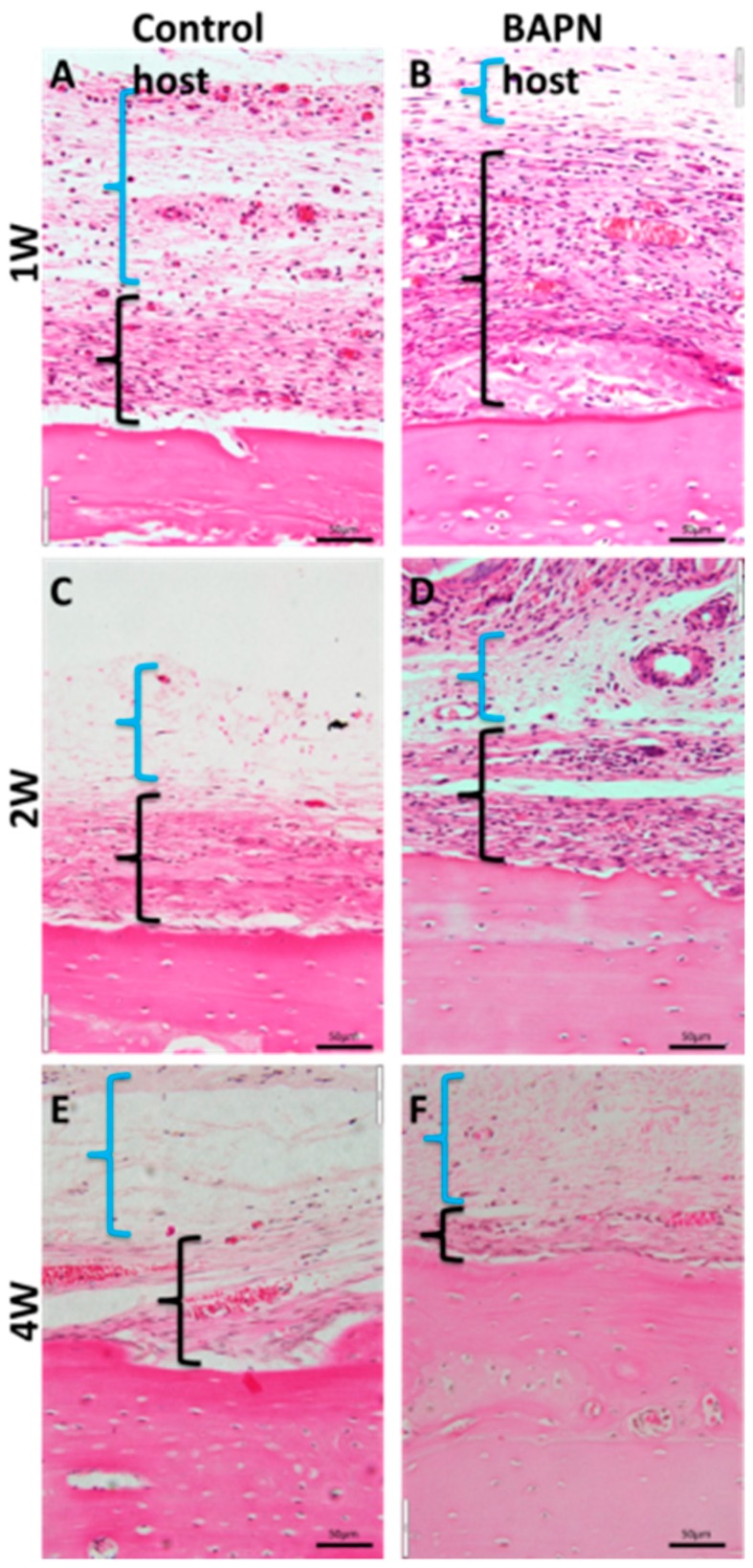
Hematoxylin and eosin staining of host periosteum surrounding transplanted bone chips at three times points. (**A**,**C**,**E**) Control host animals; (**B**,**D**,**F**) BAPN host animals. The outer fibrous layer is indicated by blue brackets. The cambium layer is indicated by black brackets. At Week 1, the cambium layer is thinner in the control host than in the BAPN host. Magnification, ×40.

HE sections showed differences in periosteal thickness among experimental groups (Figure 6). Addressing the difference in periosteal thickness seemed to depend on host condition, and not transplanted bone chip condition, representing an important finding. At Week 1, the cambium layer in control rats was thinner with fewer cells (Figure 6A) compared to BAPN rats (Figure 6B). By Week 2, both control and BAPN cambium layers showed similar thickness (Figure 6C,D). In Week 4, cambium layer thickness was reduced in both groups (Figure 6E,F).

### 3.4. Bone Formation and Osteoclastic Activity Associated with the Host

**Figure 7:**
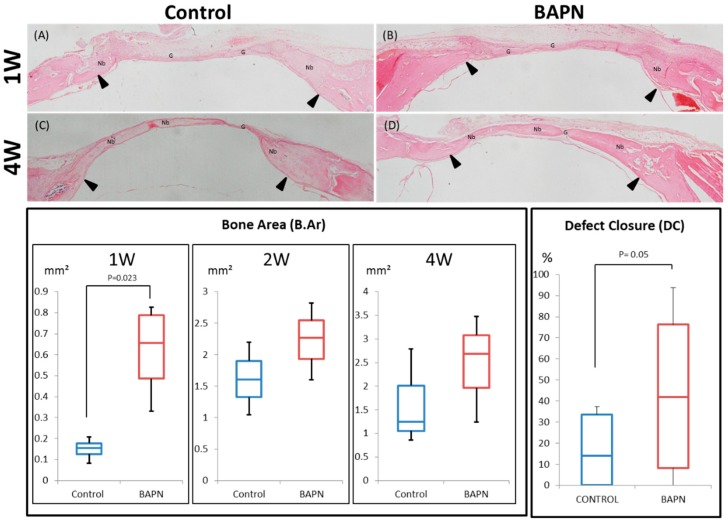
Hematoxylin and eosin staining of the gap area in both groups at Week 4 and B.Ar and DC histomorphometry in box plots. (**A**,**C**) Control; (**B**,**D**) BAPN. Black arrowheads indicate the original gap area. BAPN group tends to show higher B.Ar than control at all time points, with a significant difference evident at Week 1. BAPN DC ratio by Week 4 was significantly higher than in control. Nb: New bone; G: Gap. Magnification, ×4.

**Figure 8:**
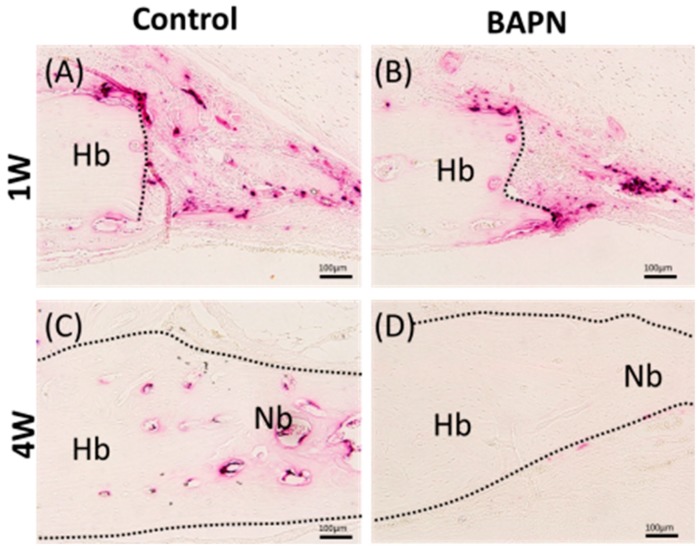
Osteoclast activity represented by TRAP staining at the cut surface of defects at Week 1 and Week 4. (**A**) Control at Week 1; (**B**) BAPN at Week 1; (**C**) Control at Week 4; (**D**) BAPN at Week 4. Osteoclastic activity disappeared in the BAPN group at Week 4. Magnification, ×20.

Both groups showed bone formation inside the gap throughout the experimental period (Figure 7). Nonetheless, HE-stained sections showed greater bone formation in the BAPN group at Weeks 1 and 4 in comparison to the control group (Figure 7A–D). Histomorphometric quantification of the bone area and gap closure also confirmed this. The bone area was significantly higher in the BAPN group than in the control group in Week 1 (*p* = 0.023), and also tended to be higher in Weeks 2 and 4, but the difference was not significant. The ratio of defect closure was significantly higher in the BAPN group than in the control group (*p* = 0.05). Osteoclastic activity was detected through TRAP staining at the cutting bone surface (Figure 8). At Week 1, both groups showed similar levels of active osteoclasts. However, BAPN group in Week 4 showed no evidence of osteoclastic activity, while the control group did.

## 4. Discussion

Many clinicians focus on the graft material, without giving much attention to the host or recipient bone condition. However, the majority of recruited cells involved in transplanted bone graft incorporation are from the host, not the graft [4]. Attention must therefore be paid to the condition of the host environment along with the graft itself. In cases of artificial graft material, irrespective of how sophisticated the graft materials are, success cannot be achieved without the proper host environment [20].

The characteristics of bone quality are directly affected by collagen cross-links [21]. We should be aware that the manipulation of bone quality factors, such as collagen cross-links, could affect the outcomes of bone transplantation surgery. The present study represents the first histological investigation to examine the effect of collagen cross-link deficiency on bone transplantation outcomes. In the present study, we produced an animal model with collagen cross-link deficiency; in which the bone architecture of the test group was changed before bone chip transplantation surgery. The animals used here were littermates from the same species [22,23,24]. Whether this kind of cross-transplantation might result in unfavorable biological reactions has been discussed, but we did not encounter any allograft rejection throughout our experiments.

Interestingly, bone chips transplanted to collagen cross-link-deficient animals integrated with host bone around 25% better than those to control host animals, regardless of the transferred bone chip condition. This result suggests that the surrounding host environment plays the main role in the incorporation of transplanted bone, rather than the transferred bone chip condition.

Counter to our expectations, we did not find any difference within the histologic boundaries of the transplanted bone chip. Osteocyte activity and necrosis appeared similar among all transplanted bone chip conditions. Furthermore, bone formation occurred underneath the transplanted bone for all bone chips. This could mean that even a bone graft bearing collagen cross-link deficiency could allow surrounding bone formation similar to that with normal bone chips. The sclerostin secreted by osteocytes acts by antagonizing Wnt signaling to terminate the bone formation process [25]. The secretion of such protein within the boundaries of transplanted bone chips in all conditions might indicate that bone grafts, at least partly, were alive and actively contributed to the bone formation process. This could be due to appropriate short-term storage during surgery and the size of the graft would allow cells far from cutting site to survive heat or cutting pressure damage.

Cambium layer cells were more abundant in collagen cross-link-deficient host animals in Week 1. The cambium layer serves as a reservoir of undifferentiated progenitor cells that differentiate into chondrogenic and osteoblastic cell lineages [26]. This could mean that collagen cross-link-deficient host animals had access to a bigger source of bone-forming cells. It is important to highlight that the periosteum originates from the host and the reaction of periosteum followed host condition, not transplanted bone condition. In addition, periosteum was completely removed before harvesting and transplantation. The new covering periosteum thus originated entirely from the host animal.

The osteoclastic activity associated with bone remodeling stage disappeared earlier in BAPN rats than in control rats, potentially reflecting higher bone cell activity. Nevertheless, a higher ability to form or remodel bone tissue does not necessarily mean a good quality of newly formed bone. In fact, research has established that a deficiency in collagen cross-links results in poor mechanical properties of the bone [6,21,27]. However, no effects on bone mineral density have been identified [10].

In the literature, BAPN treatment has been reported to result in high activity of bone cells in vitro [10,11]. In vitro experiments of other researchers have yielded contradicting results [13,28]. Our findings supported the analogy of highly active bone forming cells. In the experiment by Ida et al., the BAPN was washed from cultured cells to ensure no side effects could result from the BAPN medium, and collagen cross-links were then concluded to have affected the differentiation of bone-forming cells [16]. Also, BAPN has been reported to have no toxic effects on osteoblast-like cells [29]. In our experiments, BAPN feeding was stopped 1 week prior to the time of the first sacrifice. As a result, fewer side effects against biological reactions were anticipated.

A successful bone augmentation procedure allows osteogenesis through osteoinduction and osteoconduction [30,31]. In our experiment, transplanted bone with collagen cross-link deficiency did not stop or disturb osteogenesis in surrounding tissue. Furthermore, collagen cross-link-deficient host animals were able to form and incorporate bone faster with all transplanted bone chips. At this experiment, we did not test the efficacy of bone grafts, rather we tested the role of collagen cross-links on the bone healing around bone grafts. Based on our results we have found that host bone might be more important in the healing process than the bone graft itself. Since this work is the first work to highlight the current matter, it would be great to test other types of materials (e.g., synthetic materials) using the same experimental settings.

A common misconception between clinicians is to overemphasize on the graft materials without giving enough attention to the host site, although host bone might be the main determinant of the clinical outcome. In the future, if we can manipulate the host bone biology this might give a favorable clinical outcome. Here, collagen cross-links deficiency was evaluated in both host bone and transferred bone. Nevertheless, only the host bone with CCL deficiency had an effect on the graft incorporation while the transplanted bone condition did not affect the outcome of incorporation. This paper might help in changing the old image among clinicians about the role of collagen beyond mechanical support in bone matrix. Another perspective is that collagen cross-links deficiency is seen in some health conditions. Age-related changes and a decrease in collagen cross-links have been documented [32,33]. In osteoporosis, a significant reduction in enzymatic cross-links has been reported [7]. Saito et al. documented the drastic effect of diabetes mellitus in collagen cross-links [21].

A limitation of this study is that it did not assess the long term outcome of graft incorporation. Further study has to be done to address this long term outcome. From a mechanical perspective, it has been documented that CCL deficiency leads to low mechanical properties [6]. Therefore, under no circumstances, we do not favor poor collagen cross-linked bone over normal bone quality as it leads to a significant drop of mechanical properties of bone.

As mentioned above, the relationships between bone quality, collagen quality and collagen cross-links are well documented [6,10,34,35,36]. In the future, we need to develop a comprehensive assessment of pre-operative bone quality that incorporates all the parameters involved in bone quality. If we could manipulate all bone quality parameters before and after operations, we might be able to achieve highly predictable outcomes.

## 5. Conclusions

Deficiency of collagen cross-links accelerated bone formation in vivo. Incorporation of transplanted bone might depend mainly on host bone quality, rather than graft bone quality. Within the limitations of these experiments, further investigation is warranted to determine the clinical implications of these results

## Figures and Tables

**Table 1:** Ratio of union to non-union between transferred bone and host bone.

Host Animal	Transferred Chip	Week 4
Control	Control chip	75%
	BAPN chip	75%
BAPN	Control chip	100%
	BAPN chip	100%
